# Severe Gastroparesis After Ablation for Atrial Fibrillation

**DOI:** 10.7759/cureus.8610

**Published:** 2020-06-14

**Authors:** Junya Tanabe, Ayaka Shimizu, Nobuhide Watanabe, Akihiro Endo, Kazuaki Tanabe

**Affiliations:** 1 Cardiology, Shimane University Faculty of Medicine, Izumo, JPN

**Keywords:** ablation, atrial fibrillation, gastroparesis, mosapride citrate

## Abstract

A 70-year-old man was treated with catheter ablation for symptomatic paroxysmal atrial fibrillation (AF). The treatment consisted of pulmonary vein isolation and radiofrequency ablation of the cavo-tricuspid isthmus line. However, the patient started vomiting two days after ablation. Abdominal radiography and plain abdominal computed tomography revealed gastric distension and massive accumulation of food residues. Esophagogastroduodenoscopy after fasting for two days revealed no organic stricture; food residues were retained in the stomach but not in the duodenum, suggesting gastroparesis. The most likely mechanism underlying gastroparesis associated with AF ablation is collateral periesophageal vagal nerve injury. Mosapride citrate is considered effective for symptoms.

## Introduction

Catheter ablation (CA) is a widely accepted therapy for patients with symptomatic paroxysmal or persistent atrial fibrillation (AF). Pulmonary vein isolation (PVI) is the most common treatment for AF worldwide. Significant complications of CA include tamponade, stroke, phrenic nerve palsy, and pulmonary venous stenosis [1]. The major gastrointestinal complications associated with CA include atrioesophageal fistula, gastroparesis, esophageal thermal lesions and esophageal ulcers [1,2]. Gastroparesis is characterized by delayed gastric emptying in the absence of mechanical obstruction of the stomach. Here, we report a rare case of severe gastroparesis after CA for paroxysmal AF.

## Case presentation

**Figure 1:**
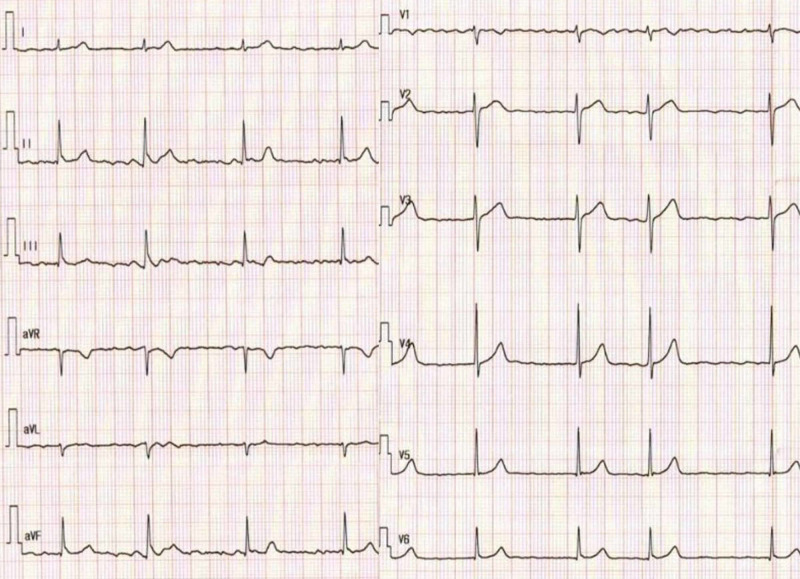
Electrocardiogram showing atrial fibrillation.

A 70-year-old man was referred to our hospital because of palpitations which occurred once every 10 days. A 12-lead electrocardiography revealed paroxysmal AF (Figure 1), and the patient requested for CA.

**Figure 2:**
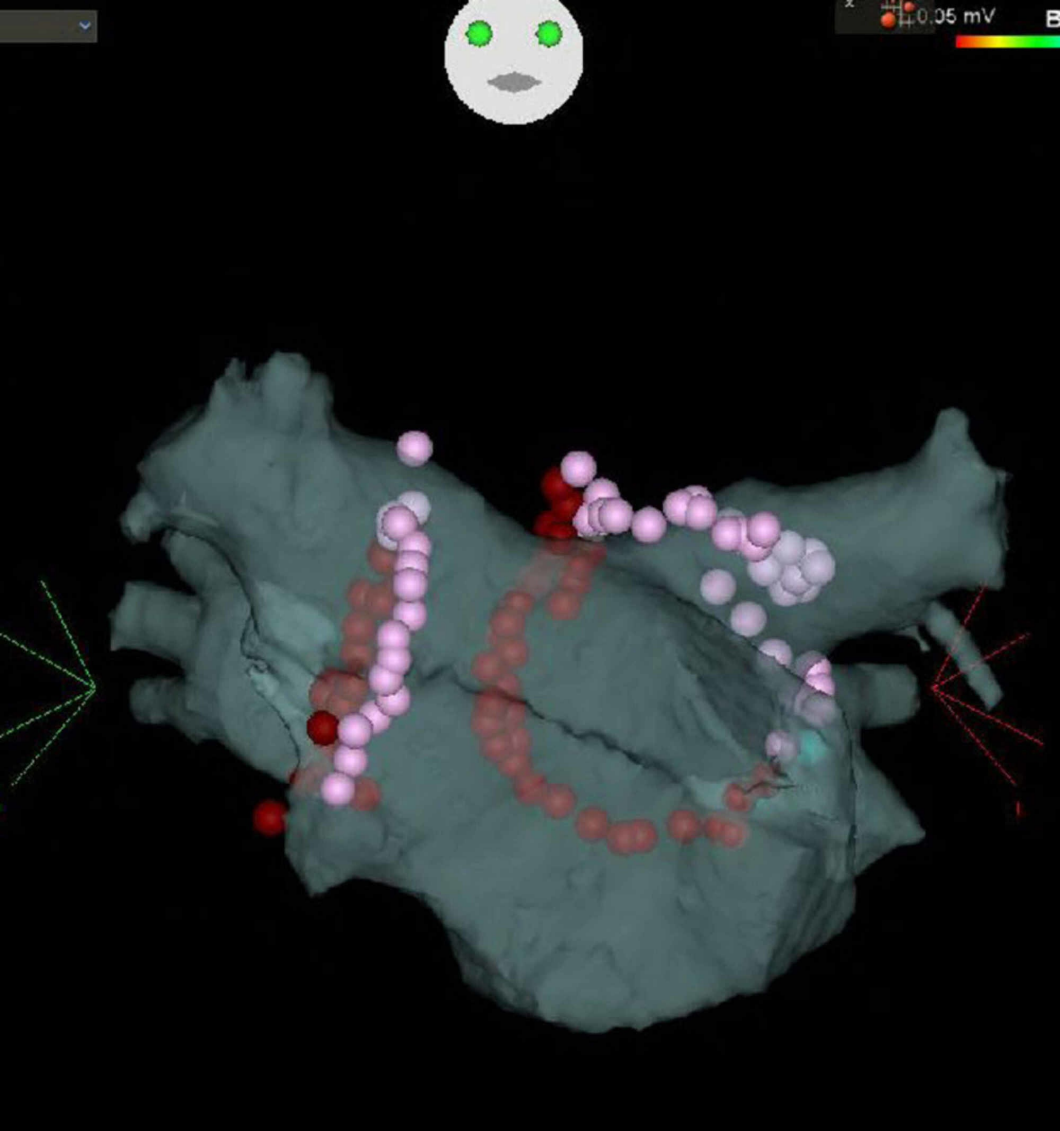
Pulmonary venous isolation with radiofrequency ablation and linear ablation of the cavo-tricuspid isthmus line were performed.

He had a history of small bowel resection due to intestinal obstruction at the age of 19 years. He had no further gastrointestinal issues since the surgery. Physical findings on admission were as follows: height, 156 cm; weight, 49 kg; blood pressure, 110/62 mmHg; heart rate, 58 beats/min with regular rhythm, and oxygen saturation, 96% on ambient air. Physical examination and blood test were unremarkable, except for a slightly elevated brain natriuretic peptide level at 26 pg/mL. Chest radiography showed a cardiothoracic ratio of 46% and no pleural effusion. Transthoracic echocardiography revealed a normal left ventricular (LV) size and LV ejection fraction of 64%. Left atrial (LA) diameter was 32 mm (normal range: 28-36 mm) and LA volume index was 36.4 mL/m^2^. The treatment consisted of PVI with radiofrequency ablation (Figure 2) and linear ablation of the cavo-tricuspid isthmus line.

**Figure 3:**
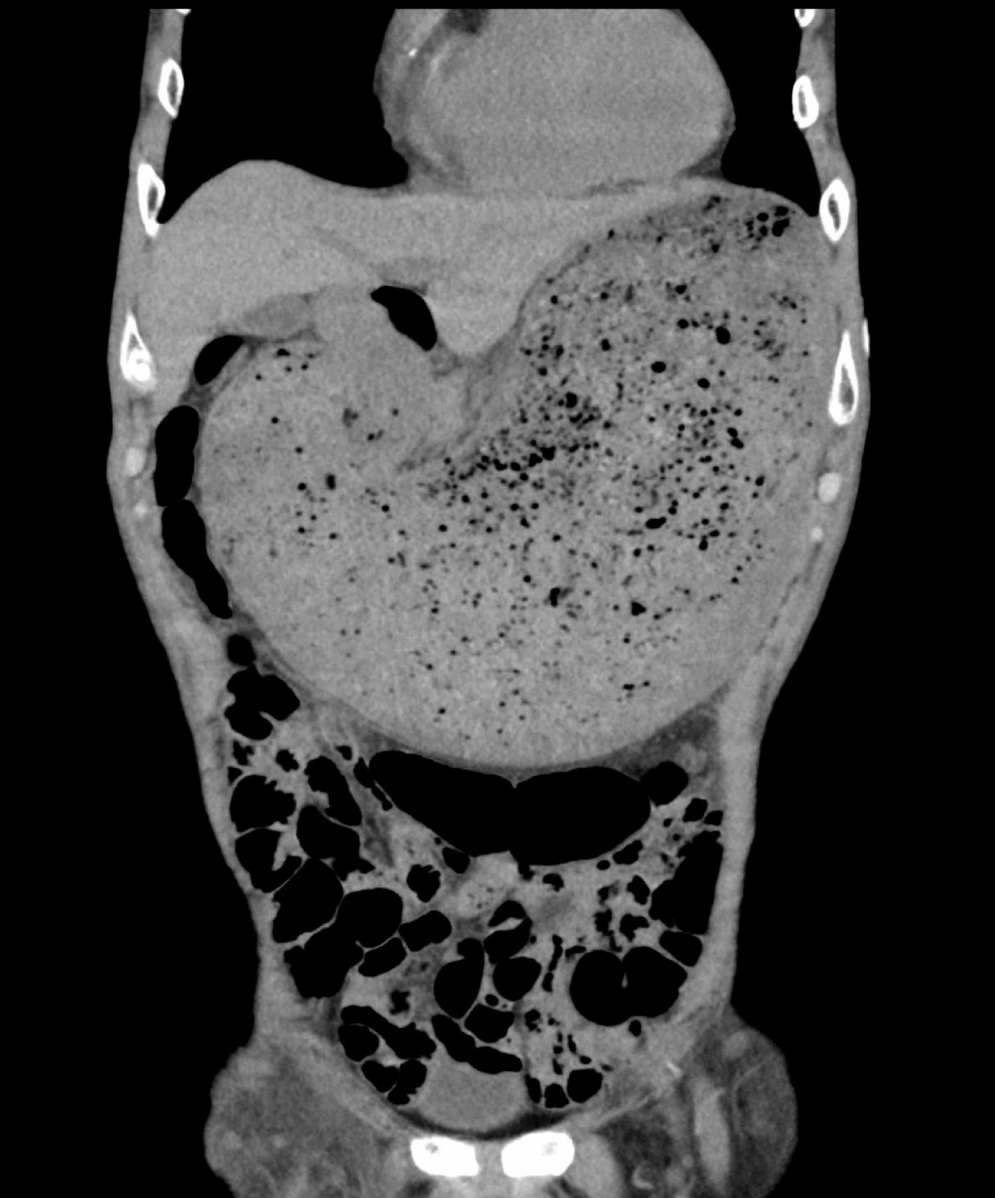
Plain abdominal computed tomography shows gastric distension and massive accumulation of food residues.

Luminal esophageal temperature monitoring was performed. The procedure was completed without major complications. Two days after CA, the patient vomited. Abdominal radiography and plain abdominal computed tomography (Figure 3) revealed gastric distension and massive accumulation of food residues.

**Figure 4:**
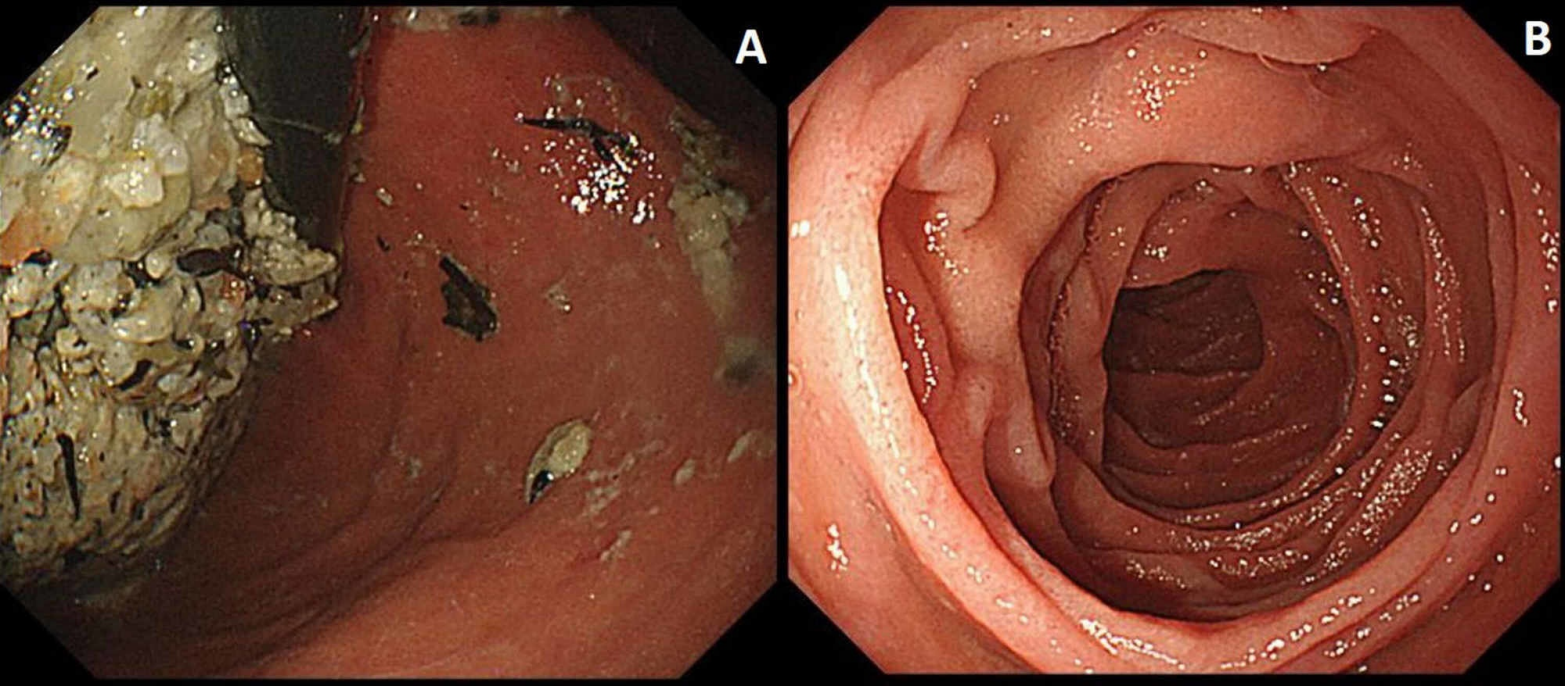
Esophagogastroduodenoscopy after fasting for two days shows no organic stricture; food residue is retained in the stomach (A). Residual food in the duodenum is not observed (B), suggesting gastroparesis.

He was not taking anticholinergic drugs. A gastric tube insertion and fasting improved the patient’s symptoms. Upper gastrointestinal endoscopy after two days of fasting revealed no organic stricture, and food residue was retained in the stomach without residual food in the duodenum, suggesting hypoperistalsis of the gastrointestinal tract (Figure 4).

The patient resumed eating, and no recurrence of abdominal symptoms was observed. Mosapride citrate was prescribed, and the patient was discharged five days after ablation therapy. His symptoms improved one week after administration of mosapride citrate. The patient continued receiving mosapride citrate, and abdominal symptoms did not recur three months after discharge.

## Discussion

Upper gastrointestinal dysmotility as a complication of AF ablation includes gastroesophageal reflux, pylorospasm, and gastric hypokinesis. Most upper gastrointestinal hypokinesis patients are asymptomatic and are often diagnosed incidentally on further testing. Upper gastrointestinal hypokinesis typically manifests within three days of catheter removal; common clinical manifestations include acute onset of reflux symptoms, dysphagia, chest pain due to esophageal hypokinesis, and abdominal distention, early satiety, weight loss, intractable nausea, and vomiting due to gastroparesis. However, there are reports of delayed upper gastrointestinal hypokinesis three months after ablation [2]. The most likely mechanism of gastroparesis associated with AF ablation is collateral periesophageal vagal injury [3]. The periesophageal vagus nerve innervating the pyloric sphincter and stomach runs close to the posterior left atrium and lower pulmonary vein [4]. Aksu et al. reported that 10% (6/58) of patients who underwent cryoablation developed symptomatic gastroparesis, whereas only 2% (1/46) of patients who underwent radiofrequency ablation developed symptomatic gastroparesis [5]. Low mean minimum temperature of the lower pulmonary vein and small left atrium diameter may be predictive factors for symptomatic gastroparesis after CA. Once the diagnosis is confirmed, oral intake should be limited to low residue diet with small fractionated meals. Patients with symptoms of gastroparesis may benefit from drugs that increase gastrointestinal motility. Rapid improvement in symptoms was observed in our patient after the administration of mosapride citrate. Mosapride citrate is a selective 5-hydroxytryptamine receptor 4 agonist, and this drug has been shown to enhance gastric empting [6]. Mosapride is thought to act directly on serotonin receptor 4 in the stomach to restore gastric peristalsis directly when nerves from the central nervous system to the stomach via the periesophageal vagal plexus are damaged. Symptoms usually resolve spontaneously in three to six months. However, mosapride citrate may promote gastric motility and lead to rapid recovery from CA-induced gastroparesis. Patients with persistent symptoms may require aggressive treatment, including botulinum toxin for pylorospasm, surgery with pacing for gastroparesis, and gastric irritation [2].

## Conclusions

We report a rare case of severe gastroparesis after CA for paroxysmal AF. Knowledge of gastrointestinal complications associated with CA include gastroparesis is important. The optimal treatment of CA-induced gastroparesis has not been established; mosapride citrate is a useful therapeutic option.
